# CXCR7 participates in CXCL12-mediated migration and homing of leukemic and normal hematopoietic cells

**DOI:** 10.1186/s13287-017-0765-1

**Published:** 2018-02-12

**Authors:** Rita de Cassia Carvalho Melo, Karla Priscila Viera Ferro, Adriana da Silva Santos Duarte, Sara Teresinha Olalla Saad

**Affiliations:** Hematology and Transfusion Medicine Center, University of Campinas/Hemocentro UNICAMP, Campinas, São Paulo Brazil

**Keywords:** CXCR7, Cell migration, Homing, Hematopoiesis, Leukemia

## Abstract

CXCR4 was the first receptor identified for CXCL12, but a second receptor, CXCR7, has also been described and its function in hematopoietic cells remains unknown. By inhibition of CXCR4 and/or CXCR7, we showed that CXCR7 participates in normal CD34^+^ and U937 cell migration and prevents downregulation of CXCR4 by CXCL12 stimulation. In addition, CXCR7 contributes to homing of acute myeloid leukemia and normal progenitor cells to the bone marrow and spleen of NOD/SCID mice. In summary, this study shows an essential role of CXCR7, together with CXCR4, in the control of normal and malignant hematopoietic cell migration and homing induced by CXCL12.

## Introduction

CXCR4 was the first receptor identified for CXCL12 [1], but a second receptor, CXCR7, has also been described [2]. CXCR7 participation in cell migration has been described mainly for malignant solid tumor cells [3], while the function of CXCR7 in hematopoietic cells remains unknown. Here we investigated the participation of CXCR7 in acute myeloid and normal progenitor cell migration through in vitro assays of chemotaxis induced by CXCL12 and homing in NOD/SCID mice.

## Results and discussion

**Fig. 1:**
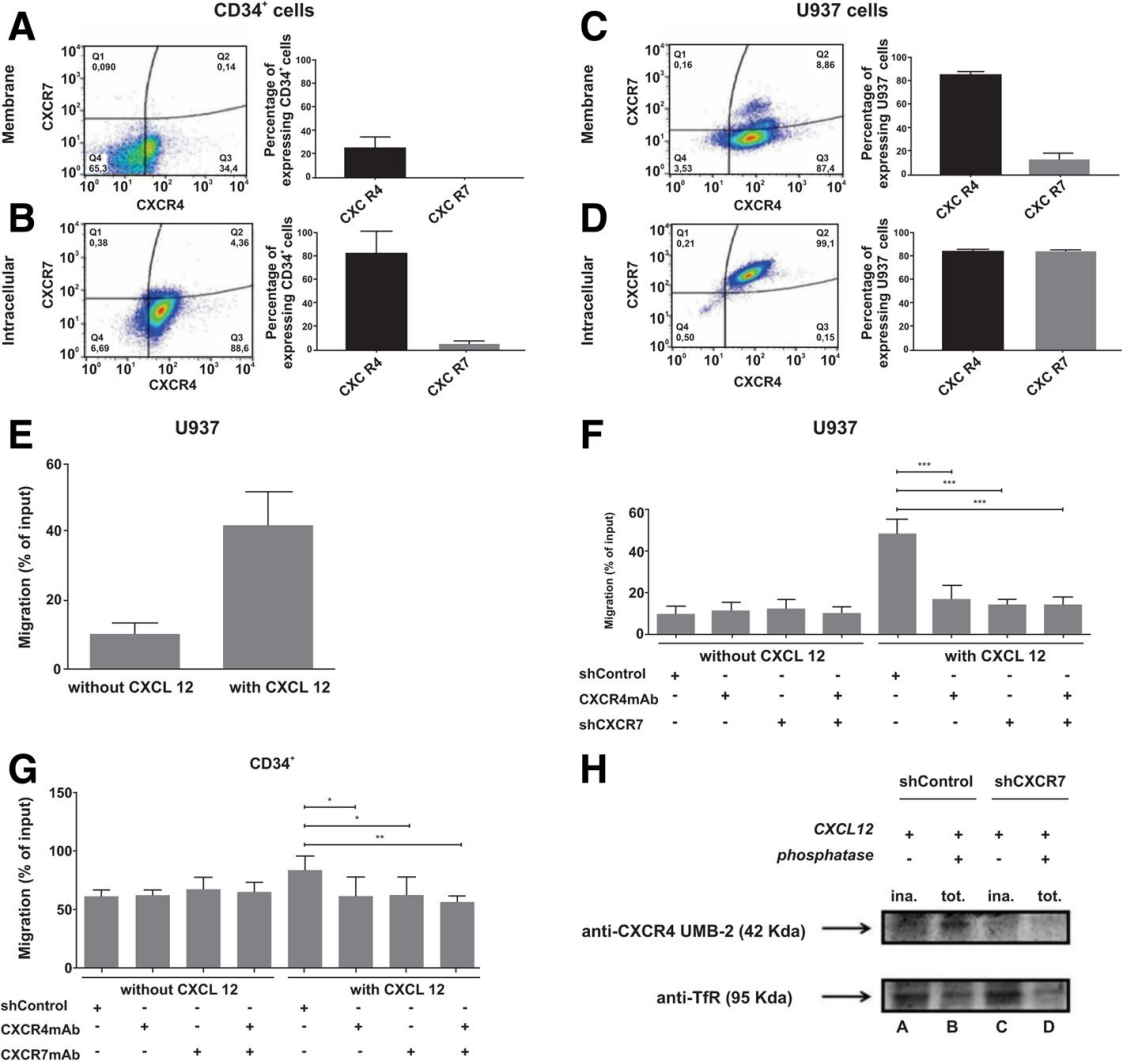
CXCR7 participates in U937 and normal CD34^+^ cell migration and prevents downregulation of CXCR4 by CXCL12 stimulation. CXCR4 and CXCR7 extracellular and intracellular expression was analyzed in CD34 (**a**, **b**) and U937 (**c**, **d**) cells on a FACScalibur flow cytometer after labeling with specific antibodies. Results are representative of one experiment of the three to five performed. Histograms show the percentage of cells expressing CXCR4 and CXCR7. The U937 cell line was chosen due to the CXCR4 and CXCR7 expression and high migration capability demonstrated towards CXCL12 (**e**). Transwell assay shows cell migration toward RPMI + 0.5% BSA, containing or not CXCL12 (200 ng/mL). The number of migrated cells (16 h for U937 and 6 h for CD34^+^) is expressed as a percentage of the input. The migration of cells was normalized to 100% ± the standard deviation of triplicates. **f** There was a significant reduction in sh*CXCR7* U937 cell migration compared to shControl U937 cells. Blocking of CXCR4 by CXCR4 mAb-clone 12G5 promoted a similar effect. Moreover, silencing of CXCR7 plus CXCR4 mAb treatment inhibited cell chemotactic capacity. **g** The same effect was observed for normal CD34^+^ cells, i.e., blockage of CXCR7 by CXCR7 mAb-clone 11G8 or CXCR4 or both receptors together also promoted a reduction in cell migration. **h** shControl and sh*CXCR7* U937 cells were stimulated with CXCL12, treated or not with phosphatase, and then labeled with anti-CXCR4 UMB-2. This antibody recognizes non-phosphorylated C-terminus. Thus, dephosphorylated samples (using phosphatase) show total CXCR4, whereas untreated aliquots show inactive CXCR4. This figure shows that, in shControl U937 cells, induction with CXCL12 caused activation of CXCR4, since the inactive form does not appear or is very low, which is characteristic of CXCR4 activation (*column A*). On the other hand, sh*CXCR7* U937 cells induced by CXCL12 showed no or low expression of total CXCR4 (*column D*), suggesting that CXCR7 is important for preventing downregulation of CXCR4 in leukemia cells. Anti-transferrin receptor (*TfR*) was used as control to ensure equal loading. Data represent three independent experiments.*p < 0.05; **p < 0.01; ***p < 0.001; one-way ANOVA and Tukey’s multiple comparison test. Error bars represent standard deviation

Using flow cytometry, we found that CXCR7 is mainly expressed in intracellular compartments of the U937 cell line and normal CD34^+^ cells. CD34^+^ cells were isolated from five different cord blood units using magnetic beads with a confirmed purity of at least 90%. We showed that CXCR7 was almost undetectable on CD34^+^ cell membranes (0.301 ± 0.105%) and a low percentage of CD34^+^ cells expressed CXCR4 on plasma membranes (24.19 ± 9.9%) (Fig. 1a); however, the percentage of CD34^+^ cells expressing intracellular CXCR4 and CXCR7 increased significantly (80.47 ± 18.76 and 5.01 ± 2.56%, respectively; Fig. 1b). Similar results were obtained for U937 cells. We observed that both receptors were mainly expressed in intracellular compartments (CXCR4 –97.68 ± 1.61%; CXCR7 –97.53 ± 3.33%; Fig. 1d) compared to surface expression (CXCR4 –89.47 ± 2.83%; CXCR7 –13.71 ± 5.59%; Fig. 1c). U937 was chosen because it is an acute myeloid leukemia (AML) cell line that expresses high levels of CXCR7 and is responsive to CXCL12, making this cell line ideal for our functional studies (Fig. 1e) Previously, CXCR7 was found scarcely expressed on the CD34^+^ surface [4, 5], but Hartmann et al. [5] observed that various specific CXCR7 blockers attenuated the ability of CXCL12 to bind to CXCR4, reducing CXCL12-mediated stimulation of integrin activation, though not chemotaxis, in T lymphocytes and CD34^+^ cells. Moreover, Torossian et al. [4] showed that CXCL12 was capable of binding to CXCR7 despite scarce expression of CXCR7 at the CD34^+^ cell surface, and blockage of this receptor inhibited Akt activation induced by CXCL12. Therefore, blocking of CXCR7 reduced cycling of CD34^+^ cells, colony formation, and survival, corroborating the hypothesis that CXCR7, with CXCR4, has a role in progenitor cells [4].

After CXCR7 silencing (lentivirus-mediated shRNA) in U937 cells, a 63% reduction in mRNA and protein levels was verified (data not shown). CXCR7 silencing did not affect the expression of CXCR4 mRNA or protein (data not shown). CXCR4 was silenced by monoclonal antibody blocking, a strategy shown to be largely effective. In normal CD34+ cells, both receptors were blocked with monoclonal antibodies.

The role of both receptors in the migration of U937 and normal CD34^+^ cells was observed by performing transwell-chemotactic assays, revealing a significant reduction in sh*CXCR7* U937 cell migration compared to shControl U937 cells (*p* < 0.001). CXCR4 blocking by mAb also reduced cell migration (*p* < 0.001). Moreover, CXCR7 silencing plus CXCR4 mAb treatment further inhibited cell chemotactic capacity (*p* < 0.001; Fig. 1f). Reduction in migration was also observed for normal CD34^+^ cells with blocked CXCR7 (*p* < 0.05) or CXCR4 (*p* < 0.05) or both receptors together (*p* < 0.01) (Fig. 1g), thus suggesting that CXCR7 contributes to migration towards CXCL12. Previous data, concurring with our results, showed the importance of this receptor in migration of T-cell acute leukemia [2] as well as AML cell lines HL60, THP-1, and U937 [6]. Kim et al. [7] performed an in vitro assay, allowing migration of U937 cells, inhibited for CXCR7, against a gradient of CXCL12 and found no significant differences compared to control cells; however, this divergent result may be explained by the short migration time (4 vs 16 h) used in their experiment. Comparing our results to those of Kim’s group, a migration of 16 h evidently led to a more pronounced and suitable result and we are thusconfident that CXCR7, together with CXCR4, controls migration of acute myeloid cells. In addition to migration, we showed that CXCR7 contributes to homing AML and progenitor cells to the bone marrow and spleen, corroborating other studies [8]. Confirming the results of Kim et al, CXCR7inhibition did not modify proliferation or apoptosis of U937 and CD34+ cells (data not shown).

We next examined the influence of CXCR7 on downregulation of CXCR4 in U937 cells induced by CXCL12. Blots were reacted with anti-CXCR4 antibody UMB-2, which recognizes the non-phosphorylated C-terminal epitope 343-352, which undergoes S346/347-phosphorylation upon CXCL12 stimulation. Thus, UMB-2 detects inactive CXCR4 in non-dephosphorylated and total CXCR4 in dephosphorylated samples. Lysate from shControl U937, induced by CXCL12, showed a difference between non-dephosphorylated and dephosphorylated aliquots, characteristic of CXCR4 activation (Fig. 1h, first and second lanes). However, *shCXCR7 U937* showed a total CXCR4 decrease when compared with shControl U937 cells (Fig. 1h, third and fourth lanes), suggesting that CXCR7 is important to prevent CXCR4 downregulation in leukemia cells. Thus, we conclude that CXCR7 prevents downregulation of CXCR4 by CXCL12 stimulation.

**Fig. 2:**
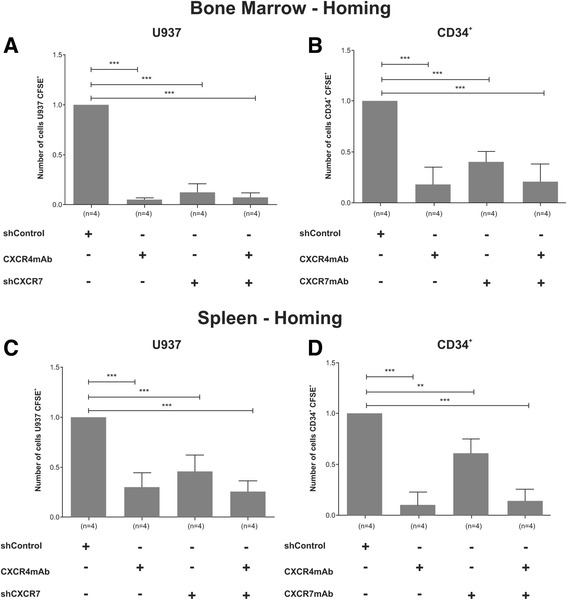
CXCR7 inhibition reduced homing of U937 and normal CD34^+^ cells to hematopoietic organs. U937 and CD34^+^ cells in which CXCR7 and/or CXCR4 were inhibited were labeled with CFSE and then injected into the tail vein of sub-lethally irradiated (3.75 Gy) female NOD/SCID mice. Bone marrow and spleen were harvested and analyzed by flow cytometry for CFSE^+^ cells 16 h after transplantation. **a** The inhibition of CXCR7 by shRNA or by blocking CXCR4 using monoclonal antibody CXCR4-clone 12G5 reduced U937 cell homing to bone marrow. CXCR7 inhibition plus CXCR4 blocking promoted the same effect. **b** Blocking of CXCR7 by CXCR7 mAb-clone 11G8 or CXCR4 by CXCR4 mAb-clone 12G5 or both receptors together reduced the homing of CD34^+^ cells to bone marrow. Reduction of homing to spleen was also observed for U937 (**c**) and CD34^+^ (**d**) cells with inhibition of CXCR7 or CXCR4 or both receptors together. Data represent four independent experiments using two different donors. **p < 0.01; ***p < 0.001; 1-way ANOVA and Tukey’s multiple comparison test. Error bars represent standard deviation

As CXCR7 showed a role in migration of leukemia and normal CD34^+^ cells, we next evaluated the homing of U937 and CD34^+^ to the bone marrow and spleen of NOD/SCID mice, after approval from the Committee on the Ethics of Animal Experiments of the University of Campinas (permit number 2679-1). U937 cells (1 × 10^7^; shControl and sh*CXCR7*), pre-treated or not with anti-human CXCR4-blocking mAb, or 5 × 10^5^ normal CD34^+^ cells, pre-treated or not with anti-human CXCR7 and/or CXCR4-blocking mAb, were labeled with CFSE (0.5 μM; Invitrogen, Carlsbad, CA, USA) and then injected into the tail vein of 6–8-week old female NOD.CB17-*Prkdc*^*scid*^/J (NOD/SCID) mice sub-lethally irradiated (3.75 Gy) at the Instituto de Pesquisas Energéticas e Nucleares of University of São Paulo (IPEN-USP). In agreement with previous reports that investigated the time course of homing, spleen cells were harvested 16 h after the transplant and bone marrow cells were isolated from mice femurs, tibias, and humerus through bone crushing. Bone marrow and spleen cells were passed through a 0.40-μM cell strainer and red blood cells were lysed with lysis buffer solution. CFSE^+^ cell acquisition was performed using a FACScalibur Flow Cytometer (Becton–Dickinson, Franklin Lakes, NJ, USA) and analyses using BD FACSDiva software (Becton Dickinson, Franklin Lakes, NJ, USA). The number of homed shControl cells was normalized to 1 (=100%) and homed cells from other groups were counted and expressed as a percentage of homed shControl cells. Inhibition of CXCR7 and/or CXCR4 significantly reduced homing of both cells to both organs (Fig. 2). Our results indicate that CXCR7 is important for migration and retention of normal and leukemic hematopoietic cells in hematopoietic organs such as the bone marrow and spleen.

The biologic function of CXCR7 depends on tissue and organ gene expression. CXCR7 does not activate signals depending on the cell type, only mediating CXCL12 internalization and degradation, acting as a scavenger receptor in cells that signal exclusively through CXCR4 [9]. In other cells, these effects are due to CXCR4 and CXCR7, but through different signaling cascades [3]. Each receiver and different signaling pathway may result in a different effect. Finally, CXCR4 and CXCR7 may form heterodimers, modulating functions mediated through CXCL12/CXCR4 interaction [10]. We further observed that CXCR7 inhibition downregulated CXCR4 expression in AML cells, probably due to excess CXCL12 not scavenged by CXCR7 [7]. Therefore, CXCR7 may be involved in CXCL12 regulation affecting processes mediated by the CXCL12/CXCR4 axis in AML cells. These findings might be extended to normal progenitor cells. Hartmann et al. [5] described two pools of CXCR7 in CD34^+^ cells: in the first, CXCR7 was expressed on the cell surface; in the second, the expression was intracellular and associated with early endosomes, which are known to participate in CXCL12 degradation. Hartmann et al. observed that CXCR7 interfered in the ability of CXCR4 to trigger optimal CXCL12-mediated stimulation of integrin activation [5] and, as observed here, CXCR7 interfered in chemotaxis and homing of CD34^+^ cells. In summary, this study shows an essential role of CXCR7, together with CXCR4, in controlling normal and malignant hematopoietic cell migration and homing induced by CXCL12.

## Abbreviations

ACKR3: Atypical chemokine receptor 3
AML: Acute myeloid leukemia
CFSE: Carboxyfluorescein succinimidyl ester
CXCL12: C-X-C motif chemokine ligand 12
CXCR4: C-X-C chemokine receptor type 4
CXCR7: C-X-C chemokine receptor type 7
mAb: Monoclonal antibody
NOD/SCID: NOD.CB17-*Prkdc*^*scid*^/J
shRNA: Short hairpin RNA
